# Physicochemical mechanisms of protein regulation by phosphorylation

**DOI:** 10.3389/fgene.2014.00270

**Published:** 2014-08-07

**Authors:** Hafumi Nishi, Alexey Shaytan, Anna R. Panchenko

**Affiliations:** ^1^Graduate School of Medical Life Science, Yokohama City UniversityYokohama Japan; ^2^National Center for Biotechnology Information, National Library of Medicine, National Institutes of HealthBethesda, MD USA

**Keywords:** protein phosphorylation, protein–protein interactions, allosteric regulation, protein disorder, multisite phosphorylation

## Abstract

Phosphorylation offers a dynamic way to regulate protein activity and subcellular localization, which is achieved through its reversibility and fast kinetics. Adding or removing a dianionic phosphate group somewhere on a protein often changes the protein’s structural properties, its stability and dynamics. Moreover, the majority of signaling pathways involve an extensive set of protein–protein interactions, and phosphorylation can be used to regulate and modulate protein–protein binding. Losses of phosphorylation sites, as a result of disease mutations, might disrupt protein binding and deregulate signal transduction. In this paper we focus on the effects of phosphorylation on protein stability, dynamics, and binding. We describe several physico-chemical mechanisms of protein regulation through phosphorylation and pay particular attention to phosphorylation in protein complexes and phosphorylation in the context of disorder–order and order–disorder transitions. Finally we assess the role of multiple phosphorylation sites in a protein molecule, their possible cooperativity and function.

## INTRODUCTION

Cellular regulatory mechanisms provide a sensitive, specific, and robust response to external stimuli and posttranslational modifications (PTMs) play an important role in these mechanisms and control protein activity, subcellular localization, and stability (Olsen et al., 2006). Such dynamic regulation is achieved through reversibility and fast kinetics of PTMs. Recent phosphoproteomic analyses have revealed that the majority of proteins in a mammalian cell are phosphorylated (Olsen et al., 2010). In eukaryotes phosphoryl group can be attached to serine, threonine, and tyrosine residues and in prokaryotes the most commonly phosphorylated residues are histidine and aspartic acid. While majority of phospho complexes in human contain only few phosphorylation sites, some proteins have up to half of their serine, threonine, and tyrosine sites phosphorylated (Nishi et al., 2011). Overall, phosphorylated serines are the most abundant (86%), followed by threonine (12%), and tyrosine phosphorylations (2%; Olsen et al., 2006). The abundance and specificity of phosphorylation as regulatory mechanism is evident from the large number of genes (more than 500) encoding protein kinases which constitute almost 2% of human protein coding genes (Manning et al., 2002). The number of phosphatases is almost ten times smaller.

In this paper we summarize biological effects of phosphorylation which are explained through the lens of structural and dynamical changes. Below we will review a number of representative studies of computer simulations of the effects of phosphorylation on protein dynamics and stability together with experimental techniques to reveal the details and underlying mechanisms of phosphorylation events at atomistic scale.

## EFFECT OF PHOSPHORYLATION ON STRUCTURE AND DYNAMICS

### STRUCTURAL CONSEQUENCES OF PHOSPHORYLATION

Phosphoryl group is dianionic at physiological pH and can form extensive hydrogen bond networks and salt bridges with neighboring residues of the same or different chains. One of the most dominant modes of interactions between phosphoryl and other residues is the interaction with the α-helical dipole at the C-terminal main chain nitrogen to neutralize the combined effect of carbonyl dipoles (Johnson and Lewis, 2001). Another common mode of interaction is the formation of hydrogen bonds and salt bridges between the phosphate oxygens and arginine or lysine side chains. Arginine side chain usually makes stronger salt bridges with phosphorylated side chains compared to lysine whereas phosphoserine (pSer) hydrogen-bond acceptor forms more stable interactions than phosphoaspartate (pAsp) acceptor (Mandell et al., 2007). Although the strength of hydrogen bonds in general should depend on the phosphate protonation state, the latter effect was shown to be rather subtle (Mandell et al., 2007) with a more pronounced effect of protonation state on pAsp than on pSer. All things considered, adding or removing a dianionic phosphate group in a protein might considerably change its local physico-chemical properties and affect stability, kinetics, and dynamics (Johnson, 2009).

Analyses of phosphorylation in different proteins revealed the diversity and heterogeneity of its effects on protein structure (Zanzoni et al., 2011), phosphorylation can impact protein structure at local as well as global levels. A recent large-scale study compared the sets of phosphorylated and unphosphorylated protein structures and showed that phosphorylation produced local as well as global changes in structure (Xin and Radivojac, 2012). Structural changes produced by phosphorylation were the highest among other PTMs. However, according to this study, only 13% of proteins exhibited the root mean square deviation (RMSD) of 2 Å or higher between phosphorylated and unphosphorylated forms and it has been argued that phosphorylation in many cases might restrict the conformational flexibility of protein monomers (Xin and Radivojac, 2012; Li et al., 2013).

There were several attempts to predict structural rearrangements induced by phosphorylation or dephosphorylation events. Although accurate predictions could be made only for a few cases, such analyses allowed to pinpoint the underlying mechanisms which govern the transitions between phosphorylated and unphosphorylated states. For example, by *in silico* phosphorylating several proteins and evaluating their conformations by OPLS-AA (all atom Optimized Potentials for Liquid Simulations) force field and a Generalized Born implicit solvent model, it was shown that structures of phosphorylated regions and conformational changes induced by phosphorylation could be predicted in some cases with near-atomic accuracy compared to the actual phosphorylated conformations (Groban et al., 2006). In another study a coarse-grained model was applied to sample the conformations of nuclear factor of activated T cells (NFAT) which was phosphorylated at multiple sites. It was found that predicted changes produced by phosphorylation differed between cytoplasmic and nuclear forms of NFAT and were driven mostly by electrostatic and solvation energy contributions (Shen et al., 2005). Several cases of the effects of phosphorylation on structure and dynamics are reviewed in **Table 1**.

**Table 1:** Selected examples demonstrating the mechanisms of phosphorylation on protein structure, stability, and dynamics.

Protein name	Method	Type of induced change upon phosphorylation	Specific outcome of phosphorylation	Reference
Short peptide with serine-proline motifs	AMD	Cis/trans isomerization of proline	Favors a helix formation	Hamelberg et al. (2005)
NFAT regulatory domain	BD, CG AMH	Conformational change	Blocks an access to the nuclear localization signaling sequence	Shen et al. (2005)
Na^+^/K^+^-ATPase	MD, electrophysiology experiments	Conformational change	Affects Na^+^ binding site and modulates ion pumping	Poulsen et al. (2012)
H3 histone tail in complex with HP1	MD	Binding affinity change	Destabilizes the complex formation	Papamokos et al. (2012)
Smooth muscle myosin regulatory light chain	TR-FRET, MD	Conformational change	Shifts conformational equilibrium to an open state	Kast et al. (2010)
c-Src-kinase	US MD	Conformational change, activity change	Locks protein in an active conformation	Meng and Roux (2014)
Collagen domain protein Shc and Epidermal growth factor receptor (EGFR)	MD, MMPBSA	Conformational rearrangement of domains, binding affinity change	Causes significant rearrangement of protein domains.	Suenaga et al. (2009)
Myelin Basic Protein (MBP)	NMR, TFE-titration, MD	Conformational change, binding affinity change	Disfavors formation of amphipathic a-helix, inhibits MBP membrane interactions	Vassall et al. (2013)
H3 histone tail, checkpoint kinase 1 (Chk1), protein kinase C (PKC)	NMR, MD	Binding affinity change, phosphorylation cooperativity and crosstalk	Phosphorylation of H3 tail affects binding of Chk1 and inhibits phosphorylation of neighboring residues	Liokatis et al. (2012)
Extracellular signal-regulated kinases (ERK)	Mutagenesis, experimental catalytic activity measurement, MD, TMD	Influence of phosphomutations on autophosphorylation	Phosphomimic mutations affect activation through autophosphorylation and folding of homodimerization interface	Barr et al. (2011)

*MD, molecular dynamics; BD, Brownian dynamics with implicit solvent; AMD, accelerated molecular dynamics; CG AMH, coarse-grain modeling with associative memory Hamiltonian; TR-FRET, time-resolved fluorescence energy transfer experiments; US MD, umbrella sampling molecular dynamics; TMD, targeted molecular dynamics; MM-PBSA, molecular mechanics Poisson–Boltzmann surface area method; NMR, nuclear magnetic resonance spectroscopy.*

### COUPLING BETWEEN PHOSPHORYLATION AND PROLINE ISOMERIZATION

Interestingly, phosphorylation might not induce the structural change by itself but rather may serve as a recognition site for an enzyme which catalyzes the conformational switch. A classical example of such mechanism is proline-directed phosphorylation which occurs on serine or threonine residues preceding proline (Lu et al., 2002). This mechanism of regulation involves specific peptidyl–prolyl *cis*/*trans* isomerase Pin1 (Lu et al., 1996) which recognizes phosphorylated Ser/Thr-Pro motif and catalyzes *cis/trans* isomerization of phosphorylated Ser/Thr-Pro bonds. Phosphorylation dramatically slows down the uncatalyzed isomerization rate of Ser/Thr-Pro bonds, while rendering them inappropriate for the action of general peptidyl–prolyl *cis*/*trans* isomerases (Yaffe et al., 1997). This complex interplay of changes introduced by phosphorylation in relation to isomerization may affect dynamics and reaction kinetics of processes involved in timing and duration of cellular response. With the help of accelerated molecular dynamics (MD) simulations, it was elegantly demonstrated in molecular details how serine phosphorylation in Ser-Pro motifs may shift the equilibrium between *cis* and *trans* proline isoforms and consequently slow down the rate of isomerization (Hamelberg et al., 2005). The authors found that isomerization of the omega-bond of proline is asymmetric and strongly depends on the psi-backbone angle of proline whereas phosphorylation might favor the α-helical backbone conformation.

### ALLOSTERIC REGULATION BY PHOSPHORYLATION AND DISORDER

Phosphorylation may trigger the transitions between conformations with different activity and/or binding specificity leading to activation or deactivation of a protein (Dou et al., 2012; Kales et al., 2012). It can play a role of covalently attached allosteric effector which induces local changes at first which may propagate thereafter into larger tertiary or quaternary structure rearrangements (Nussinov et al., 2012). One of the classical examples of a protein with large conformational changes produced by phosphorylation is glycogen phosphorylase which exists as a homodimer in inactive T state and as a tetramer in an active R state. This transition is allosterically controlled by phosphorylation of only one residue Ser14 (Johnson, 1992). Catalytic sites of glycogen phosphorylase are buried and are not solvent accessible in inactive form. Phosphorylation of Ser14 leads to large conformational movements displacing protein N-terminal region by almost 50 Å so that some intrachain contacts of Ser14 can be replaced with the contacts between pSer14 and arginine of another identical chain in a homodimer. The change in a dimer binding mode causes reconfiguration of the catalytic site and a subsequent activation of glycogen phosphorylase. Recently the model was proposed which tried to explain the allosteric coupling between phosphorylation and allostery (Mitternacht and Berezovsky, 2011). In the case of glycogen phosphorylase the authors found that the active sites had very high allosteric coupling via so-called binding leverage mechanism with those sites where the unphosphorylated N-terminal segment binds.

Another two examples illustrate the mechanism of allosteric regulation of protein activity through the coupled interplay between phosphorylation and disorder–order transitions. Activation of myosin in smooth muscle depends on the phosphorylation of regulatory light chain (RLC). In the unphosphorylated state myosin is auto-inhibited by interactions between the two catalytic domains, while phosphorylation of RLC at Ser19, which is rather distant from catalytic domain, disrupts these interactions and relieves the inhibition (Sellers, 1985). The complete mechanism by which phosphorylation of RLC activates myosin is still not known, but a series of combined experimental (EPR, TR-FRET) and MD studies were able to elucidate first steps in a cascade of conformational transitions (Nelson et al., 2005; Espinoza-Fonseca et al., 2007, 2008; Kast et al., 2010). In particular, upon phosphorylation little change in the direct vicinity of phosphorylation site is seen, while the α-helical content in region Lys11–Ala17 increases dramatically. This finding revealed a disorder–order transition induced by phosphorylation, and corroborates published experimental data on site-directed spin labeling (Nelson et al., 2005). The thermodynamic and structural basis of this phosphorylation-induced disorder–order transitions were further studied (Espinoza-Fonseca et al., 2008) and revealed a delicate balance between the gain in enthalpy due to electrostatic interactions and loss in entropy due to constraining the conformational dynamics of positively charged residues upon phosphorylation.

An interesting mechanism where phosphorylation inhibits disorder–order transition was reported for myelin basic protein (MBP), which includes a proline rich peptide containing two Thr-Pro motifs (-TPRTPPPS-) and an adjacent amphipathic a-helix which can bind to membrane (Vassall et al., 2013). Both Thr-Pro motifs can be phosphorylated by mitogen-activated protein kinases. Using a combination of NMR spectroscopy, circular dichroism spectroscopy, trifluoroethanol-titration and MD simulations the authors investigated the structure of α-helical and proline-rich regions and the effects of phosphorylation on their conformation. It was found that phosphorylation on one or both sites impedes the formation of the neighboring amphipathic α-helix. This supports the hypothesis that structure of the membrane anchoring α-helix is disrupted upon phosphorylation and thus regulates the association of MBP with the membrane. In addition, the proline-rich region may adopt PPII structure near the lipid interface when the MPB is anchored to the membrane via amphipathic helix.

## PHOSPHORYLATION IN PROTEIN–PROTEIN BINDING

### PHOSPHORYLATION ON INTERFACES MODULATES PROTEIN–PROTEIN BINDING

Many cellular control mechanisms operate at the level of protein–protein interactions, and main signaling pathways involve dense networks of protein–protein interactions and phosphorylation events. Phosphorylation may not only trigger the transitions between different conformation states of one protein but in some cases may modulate transitions between different conformations or oligomeric states in homooligomeric and heterooligomeric complexes and might represent an important mechanism for regulation of protein activity (Randez-Gil et al., 1998; Jia-Lin Ma and Stern, 2008; Hashimoto et al., 2011). Recently Nishi et al. performed a comprehensive analysis of phosphorylation sites on protein–protein binding interfaces (Nishi et al., 2011). They mapped experimentally identified phosphorylation sites onto crystal structures of human homo- and hetero-oligomers and showed that protein interfaces of transient homo- and hetero-oligomers are statistically enriched with phosphorylation sites compared to non-interfacial protein surface sites. The authors found that changes in binding affinity produced by substitutions at phosphorylation sites on binding interfaces of heterooligomers are larger compared to other sites on interfaces. In addition, consistent with the observation that phosphosites may frequently target binding hot spots, significant association between phosphosites and binding hotspots was observed (binding hot spots were defined if substitutions of residues in these sites into alanine considerably destabilizes the complex by more than 2 kcal/mol; Bogan and Thorn, 1998; Nishi et al., 2011).

Calculation of binding energy differences upon phosphorylation showed that the majority of phosphorylation events did not affect protein–protein binding (Nishi et al., 2011). It was consistent with several experimental studies pointing to the modest effect of phosphorylation on protein stability (Murray et al., 1998; Serber and Ferrell, 2007). Even if phosphorylation does not affect complex stability, it can provide diversity in recognition patterns and offer recognition sites for binding of phosphoresidue binding domains thereby modulating binding selectivity. Phosphoresidue-binding domains are common functional modules distributed widely among cellular signaling proteins. Numerous studies have identified and investigated phosphoresidue binding domains in various proteins (Via et al., 2011; Reinhardt and Yaffe, 2013) such as SH2 and PTB domains for phosphotyrosine (Pawson et al., 2001), 14-3-3 domains for phosphorserine (Yaffe et al., 1997), and FHA domains for phosphothreonine (Durocher et al., 2000). Usually these domains contain arginine or lysine residues in their binding regions to form hydrogen bonds with phosphates, and may have neighboring residues (e.g., hydrophobic residues for phosphotyrosine) which help to recognize phosphorylated site, or any specific residues in the binding motifs (Liang and Van Doren, 2008; Johnson et al., 2010). Several cases of the effects of phosphorylation on protein–protein binding are reviewed in **Table 1**.

### REGULATION OF BINDING BY DISORDER–ORDER AND ORDER–DISORDER TRANSITIONS UPON PHOSPHORYLATION

Many proteins and protein regions are intrinsically disordered under native conditions, namely, they contain no or very little well-defined structure. Folding of disordered proteins into ordered structures may occur upon binding to their specific partners which in turn might provide high specificity even if binding affinity is low (Wright and Dyson, 1999; Sugase et al., 2007). On the other hand, a number of experimental studies on p53 (Scheinin et al., 1990), cystic fibrosis transmembrane-conductance regulator (CFTR; Bozoky et al., 2013), p27 (Yoon et al., 2012), and other proteins (Johnson, 2009) have shown that disordered regions often contain phosphorylation sites and (de)phosphorylation events can be coupled to disorder–order transitions. The first systematic study was performed on a large set of proteins trying to link disordered regions with the locations of experimental phosphorylation sites. This study found that intrinsically disordered regions were enriched in phosphorylation sites (Iakoucheva et al., 2004). Moreover, protein N- and C-terminal regions which are usually disordered often participate in binding to other proteins (Fong and Panchenko, 2010) and there are many cases where terminal regions contain multiple phosphorylation sites (Chacko et al., 2001). Functional diversity of disordered regions and their propensity for PTMs allow them to play a unique role in signaling networks where phosphorylation events might serve as switches and regulate binding events. In some cases, as was shown in the previous section, the regulation of binding might happen without invoking disordered regions while in others the regulatory mechanism might involve phosphorylation as well as disorder–order or order–disorder transitions.

Before describing specific cases of proteins involving the coupling between disorder, phosphorylation and binding, we would like to describe several studies which tried to generalize fundamental principles of such coupling in many different proteins. Mohan et al. analyzed relatively short (10–70 residues) segments called MoRFs (molecular recognition features) contained within longer disordered sequences that were structurally characterized in a complex with a larger protein (Mohan et al., 2006). It was assumed that MoRFs may undergo folding upon binding but would be disordered in their unbound state. The authors of this study applied DISorder-enhanced PHOSphorylation predictor (DisPhos) to MoRF regions and found that in 305 MoRFs of more than 12 residues long, 159 of them had potential phosphorylation sites, suggesting that phosphorylation may be a common mechanism to modulate binding. Later, another group studied an association between phosphorylation and disordered binding regions in human protein complexes using experimentally identified phosphorylation sites and disorder prediction methods (Nishi et al., 2013). They showed that disordered interface residues (corresponding to sites disordered in unbound states and structured in the complex) had the highest fraction of phosphorylation sites (25%) compared to ordered interface (8%) or disordered non-interface (18%) residues, suggesting a strong association between phosphorylation and disordered interface residues. Disorder and interfacial location were significantly linked to phosphorylation of serine and to a lesser extent to phosphorylation of threonine. Tyrosine phosphorylation was not found to be directly associated with binding through disorder, and was often observed in ordered interface regions which were not predicted to be disordered in the unbound state. The fractions of phosphorylated Ser, Thr, and Tyr in disordered interfaces were 59, 26, and 15%, respectively, and were found to be quite different from those of structured interface (28, 22, and 50%; Nishi et al., 2013).

**FIGURE 1:**
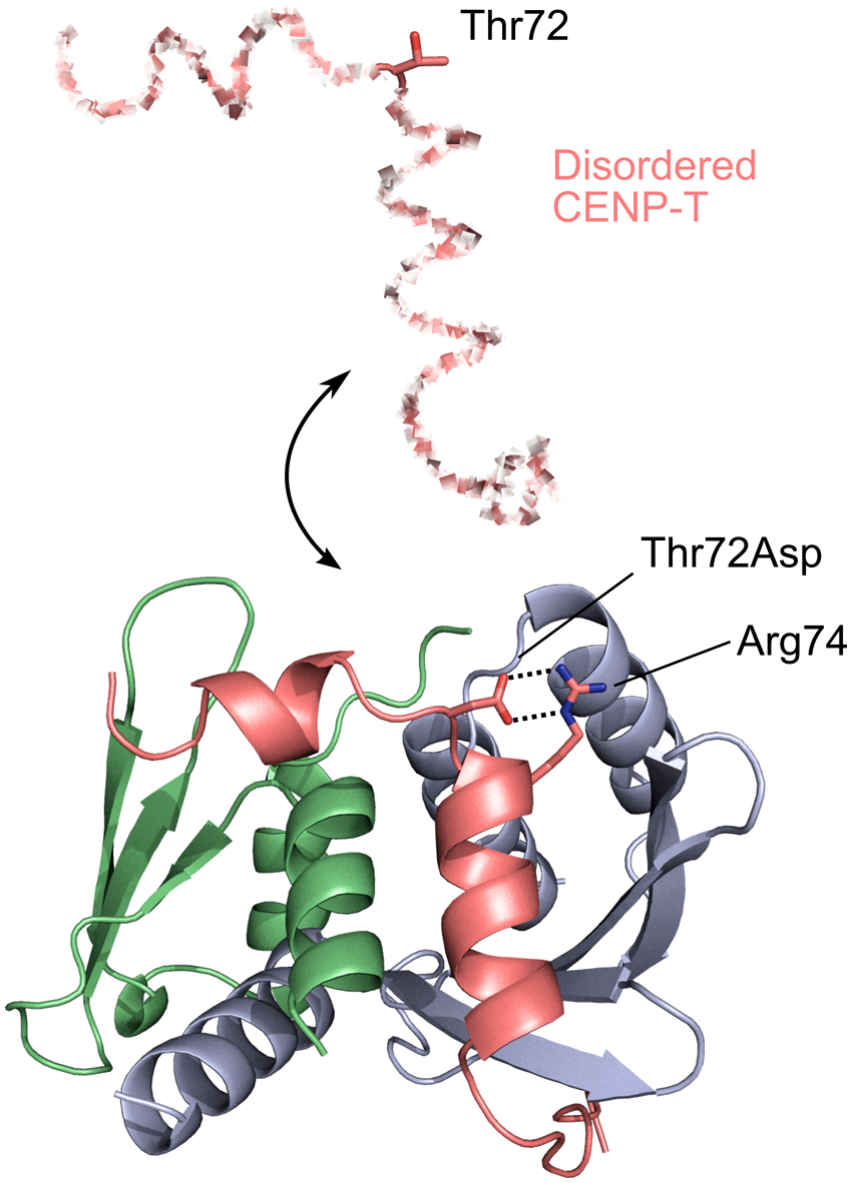
**Phosphorylation and disorder–order transition.** CENP-T-Spc24/Spc25 complex (PDBID: 3VZA). CENP-T, Spc24, and Spc25 are colored in pink, green, and blue, respectively. CENP-T has an N-terminal disordered region (shown in the dashed line) which folds when it binds to Spc24/Spc25. Phosphomimetic Thr72Asp mutant forms a salt bridge with Arg74 (shown in stick models), which enhances an interaction between CENP-T and Spc24/Spc25.

While in many disorder-involving transitions phosphorylated residues may directly regulate binding orthosterically, this is not necessarily the case. Centromere protein T (CENP-T) is an essential component of the inner kinetochore and consists of *N*-terminal disordered region and C-terminal histone-fold domain. The long disordered region is employed to bind to outer kinetochore complexes, namely, to Spc24/Spc25 subunits of Ndc80 complex (Gascoigne et al., 2011; Nishino et al., 2013). Nishino et al. (2013) revealed that phosphorylation on Thr72 of chicken CENP-T is crucial for its binding to Spc24/Spc25. The X-ray crystal structure of a complex between CENP and Spc24/Spc25 showed that binding segment comprising residues 63–93 contains two short helices, and Thr72 is located on a loop between these two helices. Interestingly, site-directed phosphomimicking mutagenesis experiments showed that Thr72Asp mutant forms a salt bridge with Arg74 on the second helix facilitating the orientation of hydrophobic residues on the second helix toward the hydrophobic patch on Spc25 partner. As a consequence, it enhances an interaction between CENP-T and Spc24/Spc25 (**Figure 1**). In addition, this phosphorylation site and salt bridge are conserved in many eukaryotic species which suggests that this mechanism is widespread for the CENP-T regulation. This example shows that phosphorylated residue can be critical for the complex formation through disorder even if it is located far away from the binding interface.

## MULTIPLE SITE PHOSPHORYLATION

### DISTRIBUTION AND FUNCTION OF MULTIPLE PHOSPHORYLATION SITES IN PROTEINS

Single protein may contain multiple phosphorylation sites. Multisite phosphorylation can expand the patterns of regulation, give more accurate modulation of conformational change (Kumar et al., 2012) and cooperatively increase binding affinity to other proteins (Ferreon et al., 2009). Large scale analyses revealed that multiple phosphorylation sites are not distributed randomly, but are often clustered on a protein (Li et al., 2009; Schweiger and Linial, 2010; Freschi et al., 2014). Namely 54% of all pSer/pThr sites are located within four residues of each other, while the tendency to form clusters is not very pronounced for pTyr sites. Clustered pSer/pThr sites are usually phosphorylated by the same kinase and clustered Ser/Thr prefer to be located in disordered regions compared to non-clustered Ser/Thr (Schweiger and Linial, 2010). Moreover, evolutionary clustered sites are 1.4 times more likely to be phosphorylated by the same kinases than expected by chance (Freschi et al., 2014).

**FIGURE 2:**
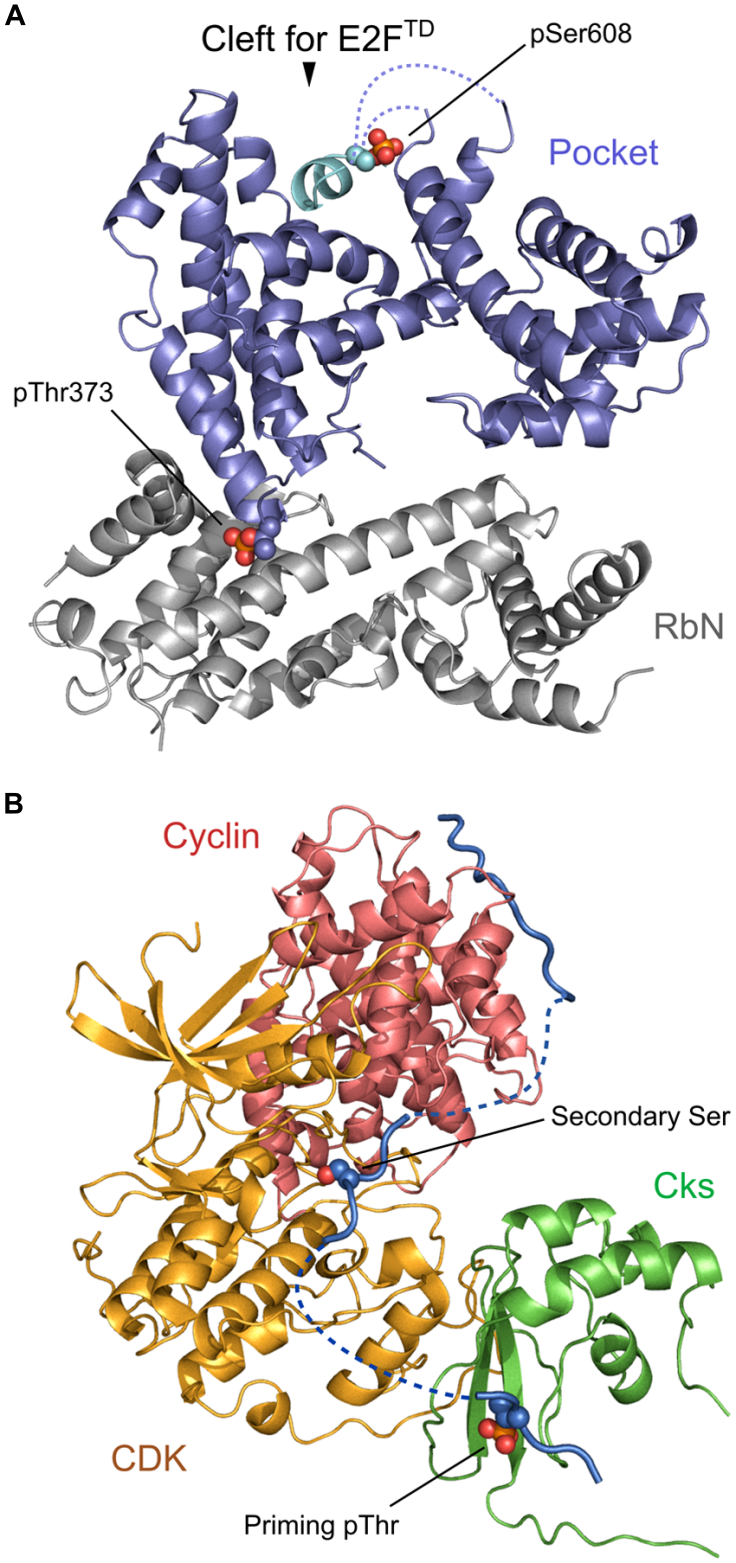
**Regulation of multisite phosphorylation. (A)** A phosphorylated model of Rb constructed based on 4ELJ and 4ELL PDB structures [as described in the previous paper (Rubin, 2013)]. Thr373 phosphorylation (shown in sphere model) induces the association between RbN (gray) and Pocket (blue) whereas Ser608 phosphorylation (shown in sphere model) allows an intra-domain loop (cyan) to directly bind to the cleft. **(B)** A model of the cyclin–Cdk–Cks1 complex with the relevant substrate peptide constructed based on 1BUH, 2CCI, and 4LPA PDB structures [as described in the previous paper (Koivomagi et al., 2013)]. Cyclin, Cdk, Cks1, and the peptide are colored in red, orange, green, and blue, respectively. Phosphorylated Thr at the priming phosphorylation site and Ser at the secondary phosphorylation sites are shown in sphere models. Structural superposition and model building was performed with Pymol.

Retinoblastoma protein (Rb) is one of the classical examples of a protein which has multiple phosphorylation sites and concerted phosphorylation patterns with very specific functional roles. Rb contains RbN, pocket, and RbC domains together with 13 different Ser/Thr phosphorylation sites that are phosphorylated by Cdk kinases. Phosphorylation sites are roughly grouped into eight clusters which mostly reside in flexible loop regions between structured regions or domains, and mediate domain–domain, domain–loop, and protein–protein interactions (Hassler et al., 2007; Burke et al., 2012). For example, Thr373 is located at the end of a flexible loop between RbN and pocket domains which do not interact if this residue is not phosphorylated. Phosphorylation of Thr373 induces large conformational changes and, as a consequence, an interaction between RbN and pocket domains, which allosterically inhibits binding of transactivation domain of E2F transcription factor (E2F^TD^) to the pocket domain (Burke et al., 2012; **Figure 2A**). Meanwhile, phosphorylation on Ser608 and Ser612 directly and orthosterically inhibits binding of E2F^TD^ to the pocket. This mechanism involves a competitive binding between E2F^TD^ and Ser608/Ser612 containing loop, namely, pSer608 stabilizes the association with the binding cleft, thereby mimicking and competing with E2F^TD^ (Burke et al., 2010; **Figure 2A**). A recent study showed that phosphorylation of Ser788 and Ser795 may also cause the inhibition of E2F^TD^ binding to the pocket by inducing the association between RbC and the pocket domain (Burke et al., 2014). This phosphorylation is additive with the effect of other preceding phosphorylations in inhibiting E2F^TD^ binding, demonstrating separate regulatory mechanisms by different phosphorylation site clusters.

### MECHANISMS OF MULTIPLE PHOSPHOSITE PROCESSING

Processes of (de)phosphorylation on multiple sites can be classified by the order of (de)phosphorylation events, which can be sequential or random. In sequential phosphorylation sites are phosphorylated in a strict order of events where phosphorylation of one site depends on the phosphorylation state of another. Sequential phosphorylation has been observed for several kinases, especially Ser/Thr kinases (Salazar and Hofer, 2009). In contrast, random phosphorylation does not require the strict order of phosphorylation events. Kinetics of (de)phosphorylation can be distinguished by the number of binding events of kinases or phosphatases. A kinase may phosphorylate all sites while staying bound to the substrate (processive mechanism) or may bind and then dissociate after each phosphorylation (distributive mechanism; Patwardhan and Miller, 2007). For example, phosphorylation of Cdc25 by Cdk1 is most likely to be random and distributive, namely, mutations on single phosphorylation sites do not preclude other phosphorylation events, and intermediate levels of Cdk1 yield partially phosphorylated Cdc25 (Lu et al., 2012). On the other hand, some kinases require “priming” phosphorylation, which automatically determines the order of phosphorylation. Known priming recognition motifs include (S/T)XXX(pS/pT) motif for GSK3 (ter Haar et al., 2001) or (S/T)XX(E/D/pS/pT) for CK2 kinases (Meggio and Pinna, 2003). Interestingly, some recent studies on human CFTR protein showed that tyrosine residue in “SYDE” motif can act as both positive and negative regulator of phosphorylation of the first serine by CK2 kinase (Cesaro et al., 2013). While “SYDE” sequence matches the CK2 canonical phosphorylation motif (SXXE), this motif is not properly phosphorylated unless tyrosine is replaced or phosphorylated.

In sequential and processive phosphorylation, distances between phosphorylation sites can be critical to maintain the phosphorylation process. Koivomagi et al. (2013) revealed the molecular mechanism of semi-processive phosphorylation on Sic1 by cyclin–Cdk1–Cks1 complex (**Figure 2B**). Sic1 contains seven Ser/Thr sites in its N-terminal region, which are phosphorylated by Cdk1. Thr5 and Thr33 were previously identified as priming phosphorylation sites compared to downstream secondary phosphorylation sites (Thr45, Thr48, Ser69, Ser76, and Ser80). First, Sic1 binds to cyclin, which induces priming phosphorylation on Thr5 and Thr33 by Cdk1. This process is inhibited if priming Thr residues are placed closer to the cyclin docking sites, indicating the importance of maintaining the proper distance between cyclin-binding motif and the phosphorylation target residues of Sic1 (Koivomagi et al., 2013). Subsequently, the phosphorylated priming sites are docked to the Cks1 pocket which in turn allows Cdk1 to access and phosphorylate downstream Ser/Thr sites which are located between the priming sites and the cyclin-binding sites. Additionally it has been shown that priming and secondary phosphorylation sites should be separated by at least 12 amino acids, otherwise the efficiency of secondary phosphorylation is greatly reduced. Overall, the authors of this paper proposed that the ability of Cdk1 to process multiple phosphorylation sites depends on spatial patterns of multiple phosphosite clusters and correct arrangements of cyclin and Cks binding elements (Koivomagi et al., 2013).

Sequential phosphorylation may also gradually increase the negative charge of a region and lead to the cooperative behavior between different phosphorylation sites. For example, phosphorylation of the Neurospora clock protein FREQUENCY (FRQ) is rate limiting for degradation and therefore crucial for circadian time keeping (Querfurth et al., 2011). This protein exists in closed and open states and in the course of the day the N-terminal domain of FRQ is sequentially phosphorylated at up to 46 sites, which increases its negative charge. As a result, the interaction with the negatively charged middle and C-terminal domains are destabilized which in turn shifts the equilibrium toward an open conformation. In an open conformation the signaling motif is exposed which targets protein for degradation.

Receptor tyrosine kinases (RTKs) represent another example. They transduce signals from the extracellular matrix to the cytoplasm of a cell and contain extracellular, transmembrane, and catalytic kinase domains and may include regulatory domains. In many cases binding of a ligand to the extracellular part induces dimerization or higher order oligomer formation and leads to the activation of intracellular kinase domain and its subsequent cross-phosphorylation. The interconversion between active and inactive states in kinases is highly regulated and kinases differ in their mechanisms of activation and inactivation (Hubbard and Miller, 2007). A key tyrosine in insulin receptor kinase domain protrudes into its active site, stabilizes inactive state and blocks access to ATP (Huse and Kuriyan, 2002) whereas tyrosines of activation loop in FGFR1 do not obstruct the ATP binding site but block the substrate binding site. Phosphorylated tyrosine can form an electrostatic contact with the basic residues, stabilize the active state of kinase and enable phosphorylation of other tyrosine residues on the C-terminal tail, which in turn mediate binding of SH2 and PTB domains of downstream signaling molecules. Phosphorylation of tyrosines happens in precise sequential order and autophosphorylation of Tyr653 in activation loop of FGFR1 increases kinase activity by 10–50-fold (Furdui et al., 2006) while subsequent phosphorylation of Tyr583, Tyr463, and Tyr585 boosts the catalytic activity up to 500-fold.

### PHOSPHORYLATION AND POST-TRANSLATIONAL MODIFICATION CROSSTALK

Post-translational modification crosstalk occurs in those cases where the presence of one modification influences the modification of another site. Phosphorylation can change the activity of proteins that regulate other types of PTMs and, as a consequence, can promote or inhibit the modification of other sites. Several studies attempted to identify crosstalk between concerted phosphosites and other PTMs by looking at PTM sites within the sequence proximity from each other and by analyzing their evolutionary conservation and functional importance (Beltrao et al., 2012; Peng et al., 2014). Some of these studies have been recently reviewed (Lothrop et al., 2013; Gajadhar and White, 2014; Venne et al., 2014).

Phosphorylation in some cases can promote subsequent ubiquitylation and the crosstalk between phosphorylation and ubiquitylation is reciprocal, namely, phosphorylation can be regulated by ubiquitylation and vice versa (Swaney et al., 2013). Interplay between phosphorylation and protein GlcNAcylation was further examined and it was shown that an increased GlcNAcylation led to lower phosphorylation at 280 phosphosites while causing an increased phosphorylation at 148 sites (Wang et al., 2008). Different patterns of PTMs may govern the interactions with different proteins; these patterns are dynamic and may respond to changes in a cellular state. In particular, it was found that majority of proteins detected in response to stimulation with epidermal growth factor (EGF) were phosphorylated on multiple sites. Moreover, various phosphosites on one protein showed different kinetics pointing to the fact that they might play different functional roles (Olsen et al., 2006).

Several comprehensive statistical studies were recently performed trying to decipher the co-evolutionary links between different types of post-translationally modified sites. It was shown that phosphorylation associates with eleven other PTM types, followed by glycosylation and acetylation (Minguez et al., 2012; Beltrao et al., 2013). In addition it was reported that the coordination between different types of PTMs may occur at the level of one subunit in a protein complex since subunits highly modified by one PTM were also enriched by other PTM type (Woodsmith et al., 2013).

Crosstalk between phosphorylation and other PTMs can be illustrated by an example of histone tail modifications that are sometimes called the “histone code.” One important modification includes histone H3 Lys9 methylation that creates a binding site for chromodomain of heterochromatin protein 1 (HP1) which plays a key role in heterochromatin formation. Adjacent residue Ser10 is a known phosphorylation site. While the ultimate mechanism of Lys9 methylation and Ser10 phosphorylation crosstalk in H3 histone is still unknown, MD simulations showed that upon phosphorylation Ser10 forms a stable salt bridge with Arg8 rather than with the positively charged Lys9. It leads to a rearrangement of tail conformation and affects the binding of HP1 to methylated H3 Lys9 (Papamokos et al., 2012).

## CONCLUSION

In this paper we reviewed the present state of the structural and biophysical studies of protein phosphorylation. Physicochemical consequences of phosphorylation are very diverse which makes it difficult to summarize and deduce general mechanisms of phosphorylation events. However, recent experimental and computational studies point to several major mechanisms for how phosphorylation may ultimately affect and modulate protein function. They include orthosterical and allosterical effects of phosphorylation on protein structure and protein–protein binding, disorder–order and order–disorder coupled transitions upon phosphorylation and, finally, cooperativity and crosstalk between multiple phosphorylation sites or other PTMs. The structural and biophysical characterization of phosphorylation crosstalk is still in its infancy but in the future it will provide important clues about mechanisms of signal propagation, integration, and separation.

## Conflict of Interest Statement

The authors declare that the research was conducted in the absence of any commercial or financial relationships that could be construed as a potential conflict of interest.
